# Effects of phenolic-rich extracts of *Clinacanthus nutans* on high fat and high cholesterol diet-induced insulin resistance

**DOI:** 10.1186/s12906-016-1049-5

**Published:** 2016-02-29

**Authors:** Nadarajan Sarega, Mustapha Umar Imam, Norhaizan Md Esa, Norhasnida Zawawi, Maznah Ismail

**Affiliations:** Institute of Bioscience, Laboratory of Molecular Biomedicine, Universiti Putra Malaysia, Serdang, Selangor 43400 Malaysia; Department of Nutrition and Dietetics, Faculty of Medicine and Health Sciences, Universiti Putra Malaysia, Serdang, Selangor 43400 Malaysia; Department of Food Science, Faculty of Food Science, Faculty of Food Science and Technology, Universiti Putra Malaysia, Serdang, Selangor 43400 Malaysia

**Keywords:** *Clinacanthus nodding*, High fat and high cholesterol diet, OGTT, Insulin resistance

## Abstract

**Background:**

*Clinacanthus nutans* is used traditionally in many parts of Asia to improve well-being, but there are limited studies on its efficacy. We explored the potential use of *C. nutans* for prevention of high fat and high cholesterol diet-(HFHC-) induced insulin resistance in rats.

**Methods:**

The leaf of *C. nutans* was extracted using water (AL extract) and methanol (AML extract), and the extracts were fed to rats alongside the HFHC diet for 7 weeks, and compared with simvastatin. Oral glucose tolerance test, and serum insulin, retinol binding protein 4 (RBP4), adiponectin and leptin were measured. Homeostatic model assessment of insulin resistance (HOMA-IR) was computed, while transcriptional regulation of hepatic insulin signaling genes was also assessed.

**Results:**

Glycemic response was higher in the HFHC group compared with the AL and AML groups, which also had lower serum RBP4, fasting glucose, insulin and HOMA-IR. Serum adiponectin levels were higher, while leptin levels were lower in the AML and AL groups compared to the HFHC group. There was upregulation of the Insulin receptor substrate, phosphotidyl inositol-3-phosphate, adiponectin receptor and leptin recetor genes, in comparison with the HFHC group.

**Conclusions:**

Overall, the results showed that the HFHC diet worsened metabolic indices and induced insulin resistance partly through transcriptional regulation of the insulin signaling genes. *C.nutans*, on the other hand, attenuated the metabolic effects and transcriptional changes induced by the HFHC diet. The results suggested that *C.nutans* may be a good source of functional ingredient for the prevention of insulin resistance.

**Electronic supplementary material:**

The online version of this article (doi:10.1186/s12906-016-1049-5) contains supplementary material, which is available to authorized users.

## Background

Hyperlipidemia is a common predicament in many societies due to changing lifestyle and food practices [1]. Previous studies have shown that the uncontrolled consumption of high fat and high cholesterol (HFHC) diet leads to insulin resistance [2, 3]. The resistance to the action of insulin can result from a variety of causes, including defects both in the receptor binding and at the post receptor levels [4]. In insulin signaling pathways, the binding of insulin to its receptor activates a series of cascade involving insulin receptor substrate (IRS) and phosphatidylinositol 3-kinase (PI3K), which are critical in insulin signaling and action [5]. One characteristic of the HFHC diet is that it causes enlargement of adipose tissue, which is a major secretory and endocrine organ, whose secreted proteins play physiological roles in metabolism. Accordingly, leptin, retinol binding protein 4 (RBP4), adiponectin and several other adipocytokines are reported to play a role in the regulation of insulin resistance and lipid metabolism [6]. Long term insulin resistance leads to increases in the risks of cardiovascular disease, diabetes mellitus and its associated complications such as diabetic nephropathy, retinopathy, neuropathy and cardiovascular disease [7].

Several pharmacological agents have been used to treat insulin resistance; however, these pharmacological agents cause significant side effects [8]. Studies have shown that natural products could offer similar or even better effects with lesser side effects [9]. *C.nutans (Burm. f.) Lindau*, commonly called *Sabah Snake Grass* or *Belalai Gajah*, is widely used in Malaysia, Thailand and Indonesia as traditional medicine and is categorized as an essential medicinal plant for primary health care by the Thai Ministry of Public Health, National Drug, and Committee [10]. *C.nutans* is reported to possess various medicinal properties including blood glucose lowering effect, alpha-glucosidase inhibition activity, antioxidant activities, anti-cancer properties and anti-inflammatory effects [11–15]. Moreover, this herb has been used traditionally to control diabetes, lower cholesterol and manage cancer. However, there is lack of scientific evidence regarding its effects. Thus, in this study, its effects on HFHC-induced insulin resistance were evaluated. Accordingly, the insulin resistance biomarkers such as serum insulin, leptin, adiponectin, retinol binding protein 4 (RBP4) and lipid profile were assayed, and the underlying transcriptomic changes induced by *C.nutans* on hepatic insulin resistance-related genes were evaluated. Furthermore, chromatographic analysis of the bioactives present in the extracts was also conducted.

## Methods

### Reagents and chemicals

General chemicals were purchased from either Sigma-Aldrich Chemical (USA) or Thermo Fisher Scientific (Massachusetts, USA). All the chemicals used in this study were of analytical reagent grade including methanol, acetic acid, acetonitrile, petroleum ether and phosphoric acid. Phenolic acid standards (Vanillic, *proto*-Catechuic acid, Cinnamic acid, Chlorogenic, Gallic, Caffeic and *p*-Coumaric) were purchashed from Sigma Aldrich Chemical (USA). Genome LabGeXP Start Kit was obtained from Beckman Coulter Inc. (USA), and the RNA isolation kit (GF-TR-100 RNA Isolation Kit) was purchased from Vivantis (Selangor, Malaysia). RCL 2 was purchased from Alphelys (Toulouse, France) and MgCl_2_ as well as DNA Taq polymerase were purchased from Thermo Fisher Scientific (Pittsburgh, PA). The fine sugar and starch powders used to make pellets were purchased from R & S Marketing Sdn. Bhd. (Malaysia), and the Palm Oil, Nespray fortified milk powder, and standard rat chow were purchased from Unilever (Malaysia), Nestle Manufacturing (Malaysia), and Specialty Feeds (TN, USA), respectively

### Collection of plant materials and sample preparation

*C.nutans* was collected on February, 2012 from YPL Herbal Farm, Taipei, Seremban, Negeri Sembilan, Malaysia. Authentication was made ​​by the botanist at the Herbarium of Biodiversity Unit, Institute of Bioscience, Universiti Putra Malaysia where the voucher specimen was deposited SK 2002/12.

### Proximate and mineral analyses of C. nutans leaf

The moisture content was determined using the official method of Association of Official Analytical Chemists [16]. A convection oven was used to dry the samples until constant weight was obtained, and the moisture content was calculated as follows:

$$ Percent\  of\  moisture = \left[1 - \left( weight\  of\  dry\  sample\ /\  weight\  of\  wet\  sample\right)\right] \times 100. $$

Furthermore, the determination of lipid content was performed following Soxtec method using Soxtec™ 2050 automated Analyzer (FOSS Analytical, Denmark), based on the official method of Association of Official Analytical Chemists [16]. Petroleum ether was used for the extraction and the fat content was obtained following the equation:

$$ Percent\  of\  fat= Weight\ \left( cup + residue\right)\ \hbox{--}\ Weight\ (cup)\Big)\ /\ \Big( weight\ (sample)\Big] \times 100x $$

Where,

Weight _(cup + residue) =_ Weight of extraction cup and residue (g)

Weight _(cup)_ = Weight of the extraction cup (g)

Weight _(sample)_ = Weight of sample (g)

The total nitrogen content in the sample was determined following the official method of the Association of Official Analytical Chemists [16]. The total nitrogen content was determined using Kjeltec™ 2200 Auto Distillation Unit (FOSS Tecator, Sweden). A nitrogen-to-protein conversion factor of 4.4 was used in the determination of protein present in the samples.

A dry ashing method was used to determine the ash content [16]. The samples were incinerated in a furnace (Furnace 62700, Barnstead/Thermolyne, IA, and USA) set at 550 °C. The remaining inorganic material was cooled, weighed and further used for the determination of mineral contents. An ash solution was prepared by dissolving the ash in 100 ml of 1 M HCl. The contents of sodium, potassium, calcium, and copper were then measured using the flame system of the Atomic Absorption Spectrophotometer (AA400, Analytik Jena AG, Jena, Germany). The results for mineral content were expressed as mg/100 g dry weight (DW).

The total carbohydrate content (%) in the samples was calculated by difference. The caloric value was calculated by the sum of the percentages of proteins and carbohydrates multiplied by a factor of 4 (Kcal/g) and total lipids multiplied by a factor of 9 (Kcal/g).

### Solvent extraction

The leaf of *C. nutans* was pulverised into fine powder using a stainless steel blender (Waring Commercial, Torrington, CT, USA) and passed through a mesh opening of 35 mm sieve. The leaf and solvent mixtures [water and aqueous methanol (80 % Methanol)] were sonicated for 60 min at 25 °C in an ultrasound water bath (Power sonic 505, Hwa Shin Technology Co., Seoul, Korea). The mixtures were then individually filtered through Whatman filter paper No. 1 and the entire extraction process was repeated twice on the residue obtained from the previous filtration process. Subsequently, solvents were removed under reduced pressure (Rotavapor R210, Buchi, Postfach, Flawil, Switzerland) followed by lyophilization (Virtis Benchtop K Freeze Dryer, SP Industries, War-Minster, PA, USA). Then, the extracts yield were calculated prior kept in - 80 °C for further analysis.

### Experimental animals

Healthy male Sprague–Dawley rats weighing about 200 g-250 g were used for the study. The animals were housed in large spacious cages. Food and water were given *ad libitum*. The animal house was well ventilated and under a 12 h light/dark cycle at the ambient temperature of 25-30 °C, throughout the experimental period. Rats were allowed to adapt to their environmental conditions for at least 10 days before the initiation of experiment. All experiments and protocols described in the study were approved by the Animal Ethics Committee (Project approval number: UPM/FPSK/PADS/BR-UUH/00484) of the Faculty of Medicine and Health Science, Universiti Putra Malaysia, Malaysia.

#### Diet preparation

The HFHC diet was formulated according to Imam et al. [17], with minor modifications. Every kg of the HFHC formulation contained 500 g ground standard rat chow, 25 g of cholesterol, 200 ml palm oil, 60 g fine sugar, 200 g Nespray® full cream milk and 50 g of starch (See Additional file 1 for diet composition). This HFHC pellet was dried in an incubator at 60 °C for 24 h, cut into small equal sized pieces and fed to the rats.

The rats were randomly divided into nine groups of seven rats each; the normal control (NC) received normal pellet, while the control group received HFHC and the STATIN groups received HFHC + oral gavage of 10 mg/kg/day simvastatin. The aqueous leaf extract (AL) and aqueous methanolic leaf extract (AML) groups were given HFHC + oral gavage of 500, 250 or 125 mg/kg/day/rat of the respective extracts.

#### Body weights, tissue collection and blood sampling

During the experiment, weekly body weights of the rats were recorded, while at the end of the experimental period (7 weeks), the animals were fasted overnight and sacrificed by dissection method. Blood (10 ml) was collected by venous puncture after an overnight fast, and centrifuged at 3000 rpm for 10 min at 4 °C to separate the serum. The serum was transferred into 1.5 ml tubes (eppendoff) and stored at −20 °C until analysis. The liver, kidney, heart, brain, spleen and lungs were excised immediately, washed with ice-cold saline, dried with filter paper, and then weighted prior to storage in formalin-free tissue fixation, RCL2 at −80 °C.

### Insulin resistance biomarkers

#### Oral glucose tolerance test (OGTT)

At the end of the intervention, OGTT was performed as described by Matsuda & DeFronzo [18] on each animal after an overnight fast, and measurements were taken with a glucometer (Roche Diagnostics, Indianapolis, IN, USA).

#### Serum insulin, glucose and homeostatic model of insulin resistance (HOMA-IR)

Serum from blood collected in plain tubes was used for measurements of insulin using the ELISA kit according to the manufacturers’ instructions. The absorbance were read on a micro plate reader (BioTeK Synergy H1 Hybrid Reader, BioTek Instruments Inc., Winooski, VT, USA) and results calculated from the standard curves; y = 0.762x – 0.143, R^2^ = 0.966. In addition, insulin resistance (IR) was assessed by the HOMA-IR, a mathematical model describing the degree of IR from fasting plasma glucose and insulin, as described previously [3].

#### Serum RBP4, adiponectin and leptin levels

Serum from blood collected in plain tubes was used for measurements of RBP4, adiponectin, and leptin using the respective ELISA kits according to the manufacturers’ instructions. Absorbances were read on BioTeK Synergy H1 Hybrid Reader (BioTek Instruments Inc., Winooski, VT, USA) at 450. The results were analyzed on www.myassays.com using four parametric test curve; adiponectin (R^2^ = 0.9954), RBP4 (R^2^ = 9969), leptin (R^2^ = 0.9916).

### Hepatic mRNA expression level

Hepatic RNA was isolated using the GF-TR-100 RNA Isolation Kit (Vivantis, Malaysia) according to the kit protocol, and primers were designed in the GenomeLabeXpress Profiler software using *Rattus norvegicus* sequence adopted from the National Center for Biotechnology Information GenBank Database (http://www.ncbi.nlm.nih.gov/nucleotide/). Genes of interest, housekeeping genes and an internal control are shown in Table 1. The forward and reverse primers had universal sequences (tags) in addition to nucleotides that were complementary to the target genes. Primers were supplied by First Base Ltd. (Selangor, Malaysia), and diluted in 1× Tris-EDTA buffer to a final concentration of 500 nM for reverse primer and 200 nM for forward primers. Then, reverse transcription and multiplex PCR of RNA samples (50 ng each) were done in an XP Thermal Cycler (BIOER Technology, Hangzhou, China) according to the kit protocol, while PCR products (1 μL each) from the above reactions were mixed with 38.5 μL of sample loading solution and 0.5 μL of DNA size standard 400 (Beckman Coulter, Inc, Miami, FL, USA) in a 96-well sample loading plate and analyzed in the GeXP machine (Beckman Coulter, Inc, Miami, FL, USA). The results from the machine were analyzed using the Fragment Analysis module of the GeXP system software and then imported into the analysis module of eXpress Profiler software. Normalization was done with GAPDH.

**Table 1:** Names, accession number and primer sequences used in the study

Gene name		Primer sequence (with universal tag)	
		Forward primer	Reverse primer
AdiponectinR2	NM_001037979	AGGTGACACTATAGAATACACTCCTGGAGAGAAGG	GTACGACTCACTATAGGGACTGAATGCTGAGTGATACAT
IRS	NM_017071	AGGTGACACTATAGAATAAGCTGGAGGAGTCTTCAT	GTACGACTCACTATAGGGAAAGGGATCTTCGCTTT
Pik3	NM_133399	AGGTGACACTATAGAATACAAGGATCTGACTTATTTCC	GTACGACTCACTATAGGGACTAACCATGCTGTTACCAA
LeptinR	NM_012596	AGGTGACACTATAGAATACAAAGTCCAGGATGACAC	GTACGACTCACTATAGGGACTTGGACAAACTCAGAATG
PPIA ^a^	NM_017101	AGGTGACACTATAGAATATTCTGTAGCTCAGGAGAGCA	GTACGACTCACTATAGGGATTGAAGGGGAATGAGGAAAA
GAPDH^a^, ^b^	NM_017008	A GGTGACACTATAGAATAATGACTCTACCCACGGCAAG	GTACGACTCACTATAGGGAAGCATCACCCCATTTGATGT
KanR ^c^			

^a^ House Keeping gene, ^b^ Normalized gene, ^c^ Internal control

Reverse transcription (RT) and PCR were done according to manufacturer’s instructions; RT reaction was at 48 °C for 1 min; 37 °C for 5 min; 42 °C for 60 min; 95 °C for 5 min, then hold at 4 °C, while PCR was as follows: initial denaturation at 95 °C for 10 min, followed by two-step cycles of 94 °C for 30 s and 55 °C for 30 s, ending in a single extension cycle of 68 °C for 1 min

### Analysis of selected phenolic compounds by HPLC-DAD

HPLC-DAD analysis was performed to identify and quantify major phenolic compounds in the leaf extracts of *C. nutans*; aqueous leaf (AL) and aqueous methanol leaf (AML) extracts. Samples were injected using an Agilent G1310A auto-sampler into an Agilent 1200 series HPLC linked with DAD 1300 diode array detector (Agilent, Stevens Creek Blvd Santa Clara, USA). Chromatographic separations were performed on a LUNA C-18 column (5 mm, 250 x 4.6 mm) (Phenomenex, Torrance, CA, USA). The solvent composition and gradient elution conditions were described previously by Chan et al. [19]. The mobile phase was composed of solvent (A) water–acetic acid (94:6 v/v, pH 2.27) and solvent (B) acetonitrile. The solvent gradient was as follows: 0–15 % B in 40 min, 15–45 % B in 40 min and 45–100 % B in 10 min. A flow rate of 0.5 ml/min was used and 20 μl of sample were injected. Samples and mobile phases were filtered through a 0.22 μm Millipore filter, type GV (Millipore, Bedford, MA) prior to HPLC injection. The standards used were Ferulic acid, PCA, Gallic acid, *p*-Coumaric, Chlorogenic acid, Vanillic acid and Caffeic acid, at the concentration of 0.1 mg/ml measured at 320 nm. The samples were analysed in triplicate and results were expressed as micrograms per gram (mg/g) of extract.

### Statistical analysis

The values were expressed as mean ± SD (*n* = 7) in each group. Differences between each group were assessed by one way analysis of variance (ANOVA) using SPSS 17 version with post hoc comparisons (according to Duncan’s multiple range test ). *P* < 0.05 was considered significant.

## Results

### Proximate analysis and mineral content

The mean values for the proximate analysis of the leaf of *C. nutans* are shown in Table 2. The major nutrient was crude carbohydrate (73.27 ± 3.14 % DW). The crude protein in the leaf was 5.16 ± 0.08 % DW, while the fat content was the lowest (2.21 ± 0.66 % DW), and the moisture content was 9.28 ± 0.40 % DW. The minerals present in the leaf are shown in Table 2. Potassium (K) was the most abundant followed by Calcium (Ca), Sodium (Na) and Copper (Cu).

**Table 2:** The proximate analysis and selected minerals of the leaf of *Clinacanthus nutans*

Nutritional value (% Dry weight)	Leaf
Crude Carbohydrate	73.27 ± 3.14
Crude protein	5.16 ± 0.08
Crude fats	2.21 ± 0.66
Moisture	9.28 ± 0.40
Ash	10.0 ± 0.20
Total Energy (KJ/100 g)	1310.68 ± 2.09
Minerals (mg/100 g DW)	Leaf
Sodium	6.78 ± 1.01
Potassium	1097.90 ± 6.93
Calcium	874.50 ± 31.25
Copper	0.26 ± 0.01

Values are expressed as percentage dry weight (% DW) for proximate analysis whereas for mineral content was expressed as mg/100 g dry weight (DW). All the values are means of three replicates and data is reported as mean ± standard deviation (*n* = 3)

#### *C. nutans* extracts slowed the rate of weight gain induced by HFHC diet

**Fig. 1:**
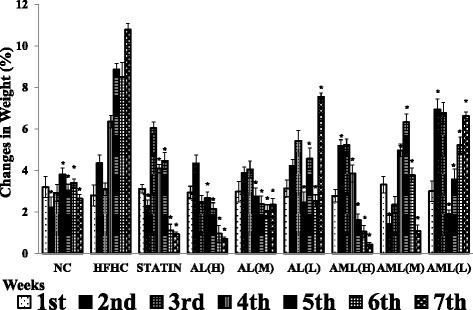
Effects of *Clinacanthus nutans* on body weight changes in high fat and high cholesterol diet-fed Sprague–Dawley rats for 7 weeks. Values are means ± SD, *n* = 7 rats/group. * *p* < 0.05 vs HFHC for each week according to Duncan’s multiple range test. NC, normal control group; HFHC, high fat and high cholesterol group; STATIN, simvastatin (10 mg/kg) group; AL (H), high dose aqueous leaf extract (500 mg/kg/day/rat) group; AL (M), medium dose aqueous leaf extract (250 mg/kg/day/rat) group; AL (L), low dose aqueous leaf extract (125 mg/kg/day/rat) group; AML (H), high dose aqueous methanolic leaf extract (500 mg/kg/day/rat) group; AML (M), medium dose aqueous methanolic leaf extract (250 mg/kg/day/rat) group; AML (L), low dose aqueous methanolic leaf extract (125 mg/kg/day/rat) group

Figure 1 shows the body weight changes throughout the experimental period. There was significant increase in body weight of the HFHC group in comparison with the NC group (*p* < 0.05). There were significant decreases in body weights of the treated groups starting from week 4, in comparison with the HFHC group (*p* < 0.05). Generally, *C. nutans* slowed the rate of weight gain dose-dependently, and by the end of the intervention period, all the treated groups had lower weights than the HFHC group.

### Organ weight

Table 3 shows the organ weights index of the liver, kidney, heart, brain, spleen and lung of the experimental rats. The HFHC group showed significantly higher weights index for the liver and kidney compared with other groups (*p* < 0.05). Additionally, the high doses of AL and AML showed lower liver weights index, which were comparable with those of the STATIN group. However, the weights index of the heart, spleen, kidney, brain and lung were not significantly different between the groups.

**Table 3:** Organ weights index of high fat and high cholesterol-fed experimental rats after 7 weeks

Organ index						
	Liver	Kidney	Heart	Brain	Spleen	Lung
NC	0.026 ± 0.001^a^	0.007 ± 0.001^a^	0.003 ± 0.002^a^	0.005 ± 0.000^a^	0.003 ± 0.001^a^	0.006 ± 0.001^a^
HFHC	0.059 ± 0.002^b^	0.005 ± 0.000^b^	0.003 ± 0.001^a^	0.005 ± 0.001^a^	0.003 ± 0.001^a^	0.005 ± 0.000^a^
STATIN	0.042 ± 0.005^c^	0.006 ± 0.001^a^	0.003 ± 0.001^a^	0.006 ± 0.001^a^	0.003 ± 0.000^a^	0.006 ± 0.001^a^
AL (H)	0.043 ± 0.004^c^	0.007 ± 0.001^a^	0.003 ± 0.001^a^	0.005 ± 0.001^a^	0.003 ± 0.001^a^	0.006 ± 0.001^a^
AL (M)	0.045 ± 0.004^c^	0.006 ± 0.001^a,b^	0.003 ± 0.001^a^	0.006 ± 0.001^a^	0.003 ± 0.000^a^	0.006 ± 0.001^a^
AL (L)	0.049 ± 0.003^c^	0.006 ± 0.000^a^	0.003 ± 0.001^a^	0.005 ± 0.000^a^	0.003 ± 0.001^a^	0.006 ± 0.001^a^
MULTI (H)	0.049 ± 0.004^c^	0.007 ± 0.001^a^	0.003 ± 0.000^a^	0.003 ± 0.002^a^	0.004 ± 0.001^a^	0.007 ± 0.001^a^
AML (M)	0.053 ± 0.006^c^	0.006 ± 0.001^a,b^	0.003 ± 0.001^a^	0.005 ± 0.001^a^	0.003 ± 0.001^a^	0.005 ± 0.001^a^
AML (L)	0.045 ± 0.003^c^	0.005 ± 0.003^a,b^	0.002 ± 0.001^a^	0.005 ± 0.000^a^	0.003 ± 0.000^a^	0.005 ± 0.001^a^

Values are means ± SD, *n* = 7 rats/group. Different superscript letters in each column indicate statistical difference (*p* < 0.05) different according to Duncan’s multiple range test. Groupings are the same as Fig. 1

### OGTT

**Fig. 2:**
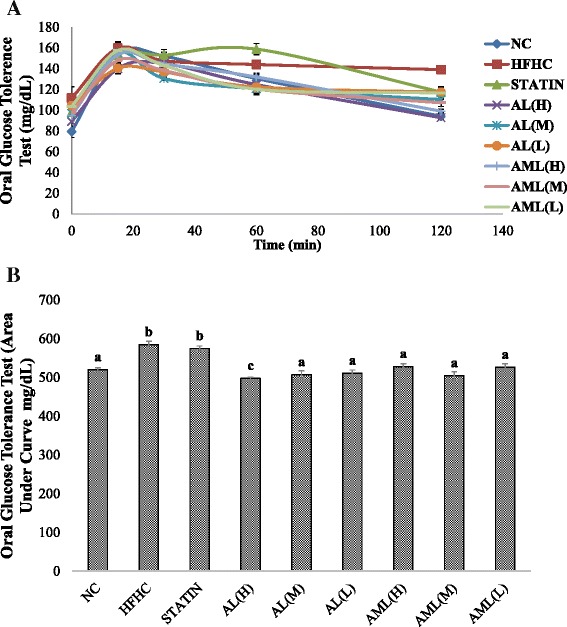
Effects of *Clinacanthus nutans* after 7 weeks of intervention on oral glucose tolerance test (**a**) and glucose area under the curve using trapeziol rule (**b**) in high fat and high cholesterol diet-fed Sprague–Dawley rats. Bars and error bars represent means ± SD (*n* = 7/group). Bars with different letters in each panel indicate statistical difference (*p* < 0.05). Groups are the same as Fig. 1

The consumption of the HFHC diet significantly increased insulin resistance biomarkers. An OGTT was performed at the end of the 7^th^ week of intervention (Fig. 2), which showed that the HFHC group had significantly higher average fasting blood glucose at baseline (before administration of the glucose load) and subsequently thereafter. The AL and AML groups showed percentage changes in dose dependent manner, from the respective base lines.

### Effects of *C.nutans* on serum insulin, glucose level, HOMA-IR and adipokines secretion

Serum insulin levels decreased in a dose dependent manner for both the AL and AML groups (*p* < 0.05) (Table 4). The fasting blood glucose levels of all the treated groups were lower in comparison with that of the HFHC group, however, only the high and medium doses of *C.nutans* showed significantly lower fasting blood glucose compared with the HFHC group (*p* < 0.05). Furthermore, the AL (H), AL (M) and AML (H) groups showed significantly improved insulin sensitivity (HOMA-IR) in comparison with the HFHC group (*p* < 0.05) (Table 4).

**Table 4:** Effects of *Clinacanthus nutans* extracts on serum insulin resistance biomarkers in high fat and high cholesterol-fed rats after 7 weeks of intervention

Groups		Insulin resistance biomarkers				
	Insulin (ng/ml)	Glucose (mmol/l)	HUMAN-IR	RBP4 (ng/ml)	Adiponectin (ng/ml)	Leptin (ng/ml)
NC	1.23 ± 0.09^a^	4.42 ± 0.34^a^	5.12 ± 1.12^a^	25.00 ± 1.72^a^	88.76 ± 16.86^a^	7.02 ± 0.81^a^
HFHC	2.97 ± 0.12^b^	6.20 ± 0.22^b^	17.36 ± 3.11^b^	56.50 ± 1.96^b^	41.54 ± 02.41^b^	3.21 ± 1.12^b^
STATIN	2.54 ± 0.11^c^	5.45 ± 1.26^b^	13.05 ± 2.09^b^	54.10 ± 4.26^b^	203.63 ± 07.43^c^	8.35 ± 0.92^a^
AL (H)	1.54 ± 0.09^d^	4.95 ± 0.36^a^	7.19 ± 1.51^a^	30.00 ± 7.32^a^	134.25 ± 05.50^d^	12.00 ± 0.41c
AL (M)	1.92 ± 0.12^e^	5.38 ± 0.57^a^	9.73 ± 1.29^c^	35.20 ± 6.68^a^	83.25 ± 11.11^a^	8.13 ± 0.51^a^
AL (L)	2.34 ± 0.10^f^	5.83 ± 0.22^b^	12.79 ± 1.90^b^	47.30 ± 12.32^b^	63.15 ± 06.07^a^	5.86 ± 1.24^a^
MULTI (H)	1.82 ± 0.10^e^	5.27 ± 0.44^a^	9.05 ± 1.51^c^	32.50 ± 8.32^a^	82.90 ± 21.20^a^	13.01 ± 1.01^c^
MULTI (M)	2.51 ± 0.12^f^	5.44 ± 0.12^c^	12.87 ± 1.43^b^	45.20 ± 2.28^c^	52.06 ± 22.40^a,b^	8.59 ± 0.73^a^
MULTI (L)	2.83 ± 0.21^g^	5.75 ± 0.32 ^b, c^	15.34 ± 1.12^b^	50.40 ± 1.07^b^	69.25 ± 15.87^a,b^	6.43 ± 0.92^a^

Values represent means ± SD (*n* = 7/group). Different superscript letters in each column indicate statistical difference (*p* < 0.05) according to Duncan’s multiple range tests. Groupings are the same as Fig. 1

There was significant elevation of RBP4 (Table 4) in the HFHC, statin and low dose *C.nutans* treated groups, while the other groups had lower levels (*p* < 0.05). HFHC feeding induced a marked decrease in the serum adiponectin level compared with the NC group. In contrast, the statin, AL (L), AL (M) and AL (H) groups had elevated adiponectin levels, and interestingly, the statin and AL (H) groups showed markedly elevated adiponectin levels (5-fold and 3-fold, respectively) in comparison with the HFHC group (*p* < 0.01). Similarly, *C.nutans* treated groups had a dose-dependent effect on the serum leptin levels.

### mRNA levels of insulin resistance-related genes

**Fig. 3:**
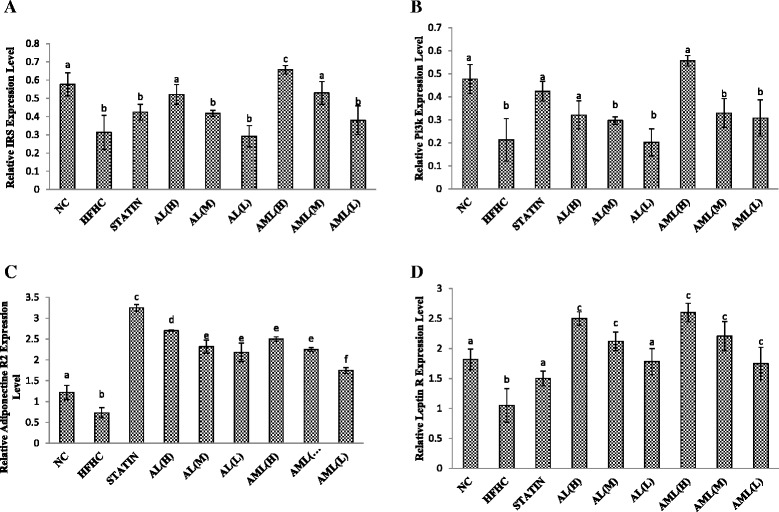
Hepatic mRNA levels of Insulin receptor substrate (IRS) (**a**), Phosphatidylinositol 3-kinase (PI3K) (**b**), Adiponectin Receptor 2 (**c**) and leptin Receptor (**d**) genes in high fat and high cholesterol diet-fed rats after 7 weeks of intervention. Bars and error bars represent means ± SD (*n* = 7/group). Bars with different letters in each panel indicate statistical difference (*p* < 0.05). Groups are the same as Fig. 1

The expressions of hepatic insulin resistance-related genes were determined to understand the effects of *C.nutans* at the transcriptomic level. As shown in Fig. 3a, the hepatic expression levels of insulin receptor substrate (IRS) were significantly elevated in rats treated with the AL(H), AML(H), AML(M) extracts compared with the untreated control (HFHC) group. Remarkably, rats treated with the AML (H) extract had the highest expression level, approximately 2 fold compared with the HFHC group (*p* < 0.01). A different trend was observed for the hepatic phosphatidylinositol-3-kinase (PI3K) expression level; only the high doses of both extracts of *C.nutans* showed significantly high expression levels and were comparable to the NC group (*p* < 0.05).

**Fig. 4:**
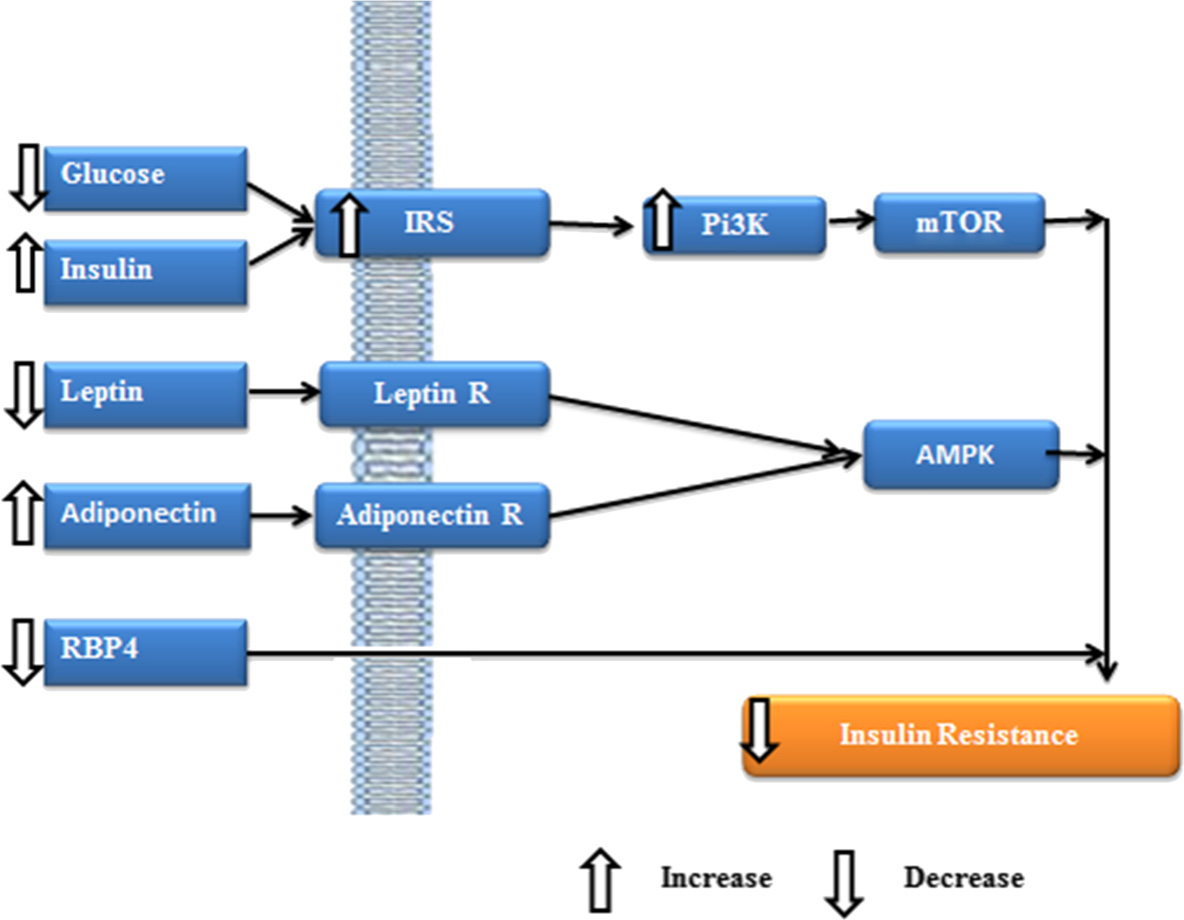
Proposed schematic diagram showing the effects of the leaf extracts of *Clincanthus nutans* on insulin resistance biomarkers

In addition, after 7 weeks of intervention, selected adipokine-related genes namely, adiponectin R and leptin R hepatic expression levels were assayed, and the results mirrored those of the ELISA tests. The adiponectin R2 expression level was suppressed in the HFHC group compared with the other groups (*p* < 0.01). Supplementation with *C.nutans* extract attenuated the effects of the HFHC diet on hepatic adiponectin R2 expression, especially at the higher doses (*p* < 0.01). On the other hand, leptin receptor expression in the HFHC group was significantly higher compared with the NC group (2 fold) (*p* < 0.01), while the treated rats showed significantly elevated leptin receptor levels, with the *C.nutans*-treated groups showing dose-dependent effects.

### Phenolic composition

Eight phenolic acids were tested, including Cinnamic acid, protocatechuic acid, Vanillic acid, Gallic acid, Caffeic acid, Ferulic acid, Chlorogenic acid and *p*-coumaric acid (Table 5). As we have recently reported [20], in both extracts, protocatechuic acid was detected to be the major phenolic acid, followed by Chlorogenic acid and trace amounts of Caffeic acid. However, *p*-Coumaric acid, Gallic acid and Vanillic acid were not detected in both of the tested extracts. Cinammic acid and Ferullic acid was detected in trace amounts in the AML extract, but not detected in the AL extract.

**Table 5:** Phenolic compositions of the extracts from the leaf of *Clinacanthus nutans*

Individual phenolic content in *C. nutans* extracts (mg/g extract)		
Phenolic Compound	Aqueous leaf	Aqueous methanol leaf
	(AL)	(AML)
Cinnamic acid	ND	0.64 ± 0.01
*Proto*-Catechuic acid	33.29 ± 0.01^a^	33.28 ± 0.01^a^
Vanillic acid	ND	ND
Gallic acid	ND	ND
Caffeic acid	5.11 ± 0.04^a^	3.62 ± 0.04^b^
Ferulic acid	ND	1.33 ± 0.02
Chlorogenic acid	22.84 ± 9.14^a^	33.38 ± 0.31^b^
*p*-coumaric	ND	ND

Data of phenolic compositions are means of three replicates and data are reported as mean ± standard deviation (*n* = 3). Different superscript letters in each row indicate statistical difference (*p* < 0.05) according to Duncan’s multiple range test. ND = non detected

## Discussion

The nutritional compositions of the leaf of *C. nutans* showed high proportion of carbohydrate, with lower amounts of ash, moisture, crude protein and crude fat. Hence, the low moisture content of *C. nutans* is indicative of its low susceptibility to microbial infection and potential long shelf-life [21]. The low protein contents may have been influenced by the use of 4.40 as the conversion factor instead of the traditional 6.25, because the latter was reported to overestimate for tropical plants or herbs [22]. The crude fat content was the lowest nutritional constituent, and may be advantageous as the caloric values are relatively low, (around 300 kcal) with added benefits for people suffering from overweight or overweight-related disease such as insulin resistance. The ash content is generally recognized as a measure of quality for the assessment of the functional properties of foods [23]. *C. nutans* contained high levels of total ash, up to 15.08 % DW, in the dried leaf. Similarly, there was a high content of minerals such as K followed by Ca, Na and Cu. The high content of K compared to Na reflects a very low Na/K ratio, which is favourable from a nutritional point of view, as diets with low Na/K ratio are associated with lower incidence of hypertension [24]. This may explain the nitric oxide (NO)-dependent hypotensive effect reported by Nwokocha et al. [25]. Cu concentrations in the leaf were relatively low (0.26 mg/100 g DW). Cu is an essential trace element needed only in minute amounts by the human body for important biochemical functions, however, as low as 10 mg per day intake may cause toxic effect [26]. Ca was found to be the second most abundant mineral element present in this plant. Therefore, *C. nutans* can be considered an appropriate dietary source of Ca to maintain the biological role of nerve transmission, muscle contraction, glandular secretion as well as mediating vascular contraction and vasodilation [27].

It has been observed that the disorders induced by high fat feeding resemble the human metabolic syndrome closely, with implications for the cardiovascular health [28]. We observed significant increase in liver weight in the HFHC group similar to the findings of Padmaja et al. [29] who demonstrated that HFHC in experimental diets will cause lipid accumulation in some organs, especially the liver. *C. nutans* attenuated the HFHC induced changes without apparent toxicity to other organs, as seen from the organ weights index in Table 3. Moreover, P’ng et al. [30] demonstrated that *C. nutans* was not toxic to the male rat liver and kidney at concentrations of up to 1800 mg/kg.

Studies have shown that there is correlation between hyperlipidemia and IR [31]. Measurements of fasting plasma glucose and insulin are widely available, and their use to calculate an index of IR (HOMA-IR) has gained wide acceptance [32]. In this study, rats fed with the HFHC diet alone showed significant worsening of IR, while administration of *C. nutans* especially at higher doses for 7 weeks caused a significant attenuation of the HFHC-induced IR. In addition, these results suggest that *C. nutans* might improve IR by normalizing the postprandial plasma glucose level as noted from the OGTT data. OGTT is one of the most critical criteria for evaluating the effectiveness of a particular compound in controlling IR and plasma glucose [33]. In the HFHC group, the elevated blood glucose levels remained high after 120 min, while in the AL and AML groups, there was significant attenuation of the blood glucose increases. Nevertheless, 120 min after glucose load, statin did not show a significant reduction in glucose level compared with the HFHC group. This study revealed that oral administration of *C. nutans* significantly improved glucose tolerance, which could be attributed to the potentiation of the insulin effect of plasma by increasing the pancreatic secretion of insulin from existing b-cells, its release from bound form or enhancements in its activity.

Cumulative researches have reported that high caloric diets lead to an increase in adipose tissue [31]. Also, evidence indicates that adipocytes, as the major cellular component of white adipose tissue, contribute to IR via adipocytokines. RBP4, adiponectin, leptin, IL-6 and TNF-α are most widely reported in this context [34]. Circulating RBP4 levels positively correlate with the degree of IR. Moreover, increased RBP4 levels are found in subjects with obesity, diabetes and cardiovascular disease [35, 36]. Intriguingly, the results of higher doses of the *C .nutans*-treated groups showed significantly lower RBP4 compared with the HFHC group, but not the statin and low doses of both extracts. Furthermore, adiponectin, believed to be produced mainly by mature adipocytes and other organs to a smaller extent, is the prototype of anti-inflammatory cytokines, and is decreased in obesity, and inversely correlated with IR, dyslipidemia, and atherosclerosis [35, 37]. In this study, the statin group showed the most beneficial biofunction followed by the AL- and AML-treated groups in dose-dependent manner. Leptin is a hormone that regulates appetite and adiposity. With the increase in adipose tissue weight, serum leptin levels also tend to decrease due to increases in lipid accumulation in various tissues of high fat diet-fed animals [38]. Moreover, the increased leptin level in serum of rats treated with *C. nutans* indicated that the lower weight in the *C.nutans*-treated rats may have contributed to this effect. The results indicate that *C. nutans* can prevent disorders related to the metabolic syndrome.

To have insights into the mechanistic basis for the regulation of the IR markers, the transcriptional regulation of genes involved in insulin signaling (IRS and PI3K) and those of selected adipokines (Adiponectin R2 and Leptin R) were evaluated. As can be recalled, any defects in the insulin signaling cascade can cause IR [4, 5]. Insulin stimulates a signaling network and the signaling axis of IRS and PI3K, which activates downstream serine/threonine kinases that regulate most of the metabolic actions of insulin, such as suppression of hepatic glucose production and activation of glucose transport in muscle and adipocyte [39]. This pathway is impaired at multiple steps through alterations in the expression levels and activities of the signaling molecules, enzymes, and transcription factors in IR caused by HFHC diet [4, 5]. Thus, compounds that are able to regulate these genes can be potentially beneficial for the management of IR-related diseases. As shown in Fig. 2 the reduced expressions of IRS and PI3K due to prolonged HFHC feeding were attenuated by *C. nutans* especially the higher doses of the extracts. Furthermore, the expressions of hepatic adiponectin R2 and leptin R genes were also modulated by treatment with *C. nutans,* in line with the changes observed in the serum adiponectin and serum leptin levels (Table 4).

The attenuation of IR biomarkers may be due to the presence of active constituents like proto-Catechuic acid, Cholorogenic acid, Caffeic acid, Cinnamic acid and Ferulic acid in the *C. nutans* extracts. Phenolic compounds are widely distributed in fruits and vegetables and are the major class of antioxidants found in plant-derived foods [40]. Proto-Catechuic acid was the major compound detected in both extracts, and may have contributed significantly to the biological activities of the plant. Scazzocchio et al. [41] demonstrated that proto-Catechuic acid possessed insulin-like effects. Chlorogenic acid may also have contributed as seen from the superior bioactivity of the AML extract with higher chlorogenic acid over the AL extract, against body weight, lipid profile and insulin resistance biomarkers. Moreover, Cholorogenic acid has been shown to regulate glucose and lipid metabolism [42]. Additionally, Cinnamic acid was detected in the AML extract, but not the AL extract. Cinnamic acid and Caffeic acid have been shown to improve glucose metabolism via modulating gluconeogenesis and glycogenesis in insulin-resistant mouse hepatocyte model [43]. Ferulic acid, on the other hand, was detected in trace amounts in both the AL and AML extract, suggesting that it may have contributed minimally to improved insulin resistance biomarkers. In general, however, based on the presence of multiple phenolics in the extracts of *C. nutans*, it is likely that synergism played a role in their overall bioactivities. We recently hypothesized that extracts with a lead compound and smaller amounts of other bioactive compounds produced superior bioactivity likely due to the synergistic effects of the multiple bioactives [44], and the same effect may have contributed to the bioactivity of the extracts used in this study. In aggregate, the data showed that the HFHC diet promoted IR through modulation of various indices, while *C. nutans* and simvastatin attenuated the HFHC-induced changes, although *C. nutans* produced better results than simvastatin. Based on the findings, we proposed the mechanistic basis for the attenuation of the HFHC-induced IR by *C.nutans* leaf extracts as shown on Fig. 4.

## Conclusions

In this study, we demonstrated that HFHC feeding will induce IR (higher OGTT, HOMA-IR, lipid leptin, RBP4 and lipid profile, and lower adiponectin levels), partly through transcriptional modulation of insulin signaling genes. *C. nutans*, however is able to prevent IR by preventing some of the transcriptional changes on insulin signaling genes induced by the HFHC likely mediated by multiple bioactive compounds including protocatechuic acid and chlorogenic acid. There is need to further evaluate the potential use of *C. nutans* in the management of IR in already established insulin-resistant conditions especially in humans and also confirm bioactive compounds responsible for the effects observed. In view of the growing interest in plant bioresources as potentially cost-effective and safer alternatives to available drugs for managing chronic diseases, this plant may potentially be a good source of functional ingredients for managing metabolic disorders like IR.

## Additional file

Additional file 1: Food Composition of the Normal Pellet and High Fat and High Cholesterol (HFHC) Diet. (DOCX 12 kb)

## Abbreviations

AL: Aqueous extract of *Clinicanthus nutans*
AML: Aqueous extract of Clinicanthus nodding
HFHC: High cholesterol and high fat
HUMAN-IR: Homeostatic model assessment of insulin resistance
AND: Insulin resistance
IRS: Insulin receptor substrate
OGTT: Oral glucose tolerance test
PI3K: Phosphotidylinositol-3-phosphate
RBP4: Retinol binding protein-4
