# Supplementary material for: Effects of phenolic-rich extracts of Clinacanthus nutans on high fat and high cholesterol diet-induced insulin resistance
Source: BMC Complement Altern Med. 2016 Feb 29;16:88. doi: 10.1186/s12906-016-1049-5 (PMC4770701; doi:10.1186/s12906-016-1049-5)
Supplement: Additional file 1: — Food Composition of the Normal Pellet and High Fat and High Cholesterol (HFHC) Diet. (DOCX 12 kb) [file 12906_2016_1049_MOESM1_ESM.docx]

**Additional file 1**

**Food Composition of the Normal Pellet and High Fat and High Cholesterol (HFHC) Diet**

| **Food classes** | **Normal Pellet (%)** | **HFHC (%)** |
| --- | --- | --- |
| Carbohydrates | 60.0 | 40.0 |
| Protein | 25.0 | 30.0 |
| Vitamin mix | 5.0 | 2.5 |
| Fats | 5.0 | 22.5 |
| Fibre | 5.0 | 2.5 |
| Cholesterol | 0.0 | 2.5 |
